# CUDA compatible GPU cards as efficient hardware accelerators for Smith-Waterman sequence alignment

**DOI:** 10.1186/1471-2105-9-S2-S10

**Published:** 2008-03-26

**Authors:** Svetlin A Manavski, Giorgio Valle

**Affiliations:** 1 CRIBI, University of Padova, Padova, Italy; 2 Elaide, Srl, Padova, Italy

## Abstract

**Background:**

Searching for similarities in protein and DNA databases has become a routine procedure in Molecular Biology. The Smith-Waterman algorithm has been available for more than 25 years. It is based on a dynamic programming approach that explores all the possible alignments between two sequences; as a result it returns the optimal local alignment. Unfortunately, the computational cost is very high, requiring a number of operations proportional to the product of the length of two sequences. Furthermore, the exponential growth of protein and DNA databases makes the Smith-Waterman algorithm unrealistic for searching similarities in large sets of sequences. For these reasons heuristic approaches such as those implemented in FASTA and BLAST tend to be preferred, allowing faster execution times at the cost of reduced sensitivity. The main motivation of our work is to exploit the huge computational power of commonly available graphic cards, to develop high performance solutions for sequence alignment.

**Results:**

In this paper we present what we believe is the fastest solution of the exact Smith-Waterman algorithm running on commodity hardware. It is implemented in the recently released CUDA programming environment by NVidia. CUDA allows direct access to the hardware primitives of the last-generation Graphics Processing Units (GPU) G80. Speeds of more than 3.5 GCUPS (Giga Cell Updates Per Second) are achieved on a workstation running two GeForce 8800 GTX. Exhaustive tests have been done to compare our implementation to SSEARCH and BLAST, running on a 3 GHz Intel Pentium IV processor. Our solution was also compared to a recently published GPU implementation and to a Single Instruction Multiple Data (SIMD) solution. These tests show that our implementation performs from 2 to 30 times faster than any other previous attempt available on commodity hardware.

**Conclusions:**

The results show that graphic cards are now sufficiently advanced to be used as efficient hardware accelerators for sequence alignment. Their performance is better than any alternative available on commodity hardware platforms. The solution presented in this paper allows large scale alignments to be performed at low cost, using the exact Smith-Waterman algorithm instead of the largely adopted heuristic approaches.

## Background

### Related works

Searching databases of DNA and protein sequences is one of the fundamental tasks in bioinformatics. The Smith-Waterman algorithm guarantees the maximal sensitivity for local sequence alignments, but it is slow. It should be further considered that biological databases are growing at a very fast exponential rate, which is greater than the rate of improvement of microprocessors. This trend results in longer time and/or more expensive hardware to manage the problem. Special-purpose hardware implementations, as for instance super-computers or field-programmable gate arrays (FPGAs) are certainly interesting options, but they tend to be very expensive and not suitable for many users.

For the above reasons, many widespread solutions running on common microprocessors now use some heuristic approaches to reduce the computational cost of sequence alignment. Thus a reduced execution time is reached at the expense of sensitivity. FASTA (Pearson and Lipman, 1988) [1] and BLAST (Altschul et al., 1997) [2] are up to 40 times faster than the best known straight forward CPU implementation of Smith-Waterman.

A number of efforts have also been made to obtain faster implementations of the Smith-Waterman algorithm on commodity hardware. Farrar [3] exploits Intel SSE2, which is the multimedia extension of the CPU. Its implementation is up to 13 times faster than SSEARCH [14] (a quasi-standard implementation of Smith-Waterman).

To our knowledge, the only previous attempt to implement Smith-Waterman on a GPU was done by W. Liu et al. (2006) [4]. Their solution relies on OpenGL that has some intrinsic limits as it is based on the graphics pipeline. Thus, a conversion of the problem to the graphical domain is needed, as well as a reverse procedure to convert back the results. Although that approach is up to 5 times faster than SSEARCH, it is considerably slower than BLAST.

In this paper we present the first solution based on commodity hardware that efficiently computes the exact Smith-Waterman alignment. It runs from 2 to 30 times faster than any previous implementation on general-purpose hardware.

### The Smith-Waterman algorithm

The Smith-Waterman algorithm is designed to find the optimal local alignment between two sequences. It was proposed by Smith and Waterman (1981) [5] and enhanced by Gotoh (1982) [6]. The alignment of two sequences is based on the computation of an alignment matrix. The number of its columns and rows is given by the number of the residues in the query and database sequences respectively. The computation is based on a substitution matrix and on a gap-penalty function.

Definitions:

• *A: a_1_a_2_a_3_….a_n_* is the first sequence,

• *B: b_1_b_2_b_3_….b_m_* is the second sequence,

• *W(a_i_, b_j_)* is the substitution matrix,

• *G_init_* and *G_ext_* are the penalties for starting and continuing a gap,

the alignment scores ending with a gap along A and B are

$$\begin{array}{l} E_{i,j}=max\left\{\begin{array}{l} E_{i,j-1}-G_{ext}\\ H_{i,j-1}-G_{init} \end{array}\right\}\\ F_{i,j}=max\left\{\begin{array}{l} F_{i-1,j}-G_{ext}\\ H_{i-1,j}-G_{init} \end{array}\right\} \end{array}$$

Finally the alignment scores of the sub-sequences *A_i_*, *B_j_* are:

$$H_{i,j}=\;\;max\;\;\left\{\begin{array}{l} 0\\ E_{i,j}\\ F_{i,j}\\ H_{i-1,j-1}-W\left(a_{i},b_{j}\right) \end{array}\right\}$$

where 1≤*i*≤*n* and 1≤*j*≤*m*. The values for E, F and H are 0 when *i*<1 and *j*<1. The maximum value of the alignment matrix gives the degree of similarity between *A* and *B*.

**Figure 1:**
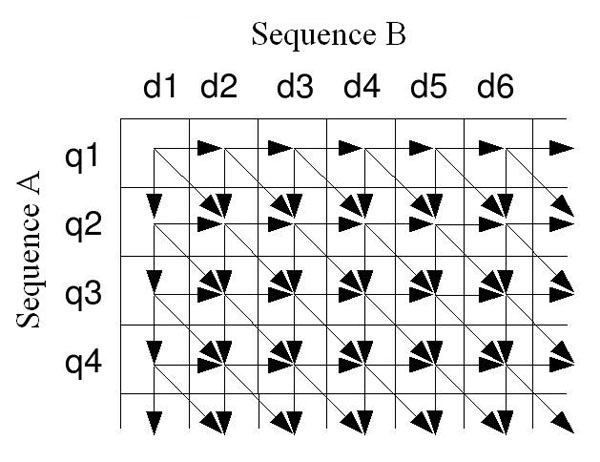
**Smith-Waterman data dependencies.** Each cell of the alignment matrix depends on the cells on the left and above it. Independent data can be found only on the same anti-diagonal.

An important point to be considered is that any cell of the alignment matrix can be computed only after the values of the left and above cells are known, as shown in Figure 1. Different cells can be simultaneously processed only if they are on the same anti-diagonal.

### CUDA programming model

The two major GPU vendors, NVidia and AMD, recently announced their new developing platforms, respectively CUDA [7] and CTM [8]. Unlike previous GPU programming models, these are proprietary approaches designed to allow a direct access to their specific graphics hardware. Therefore, there is no compatibility between the two platforms. CUDA is an extension of the C programming language; CTM is a virtual machine running proprietary assembler code. However, both platforms overcome some important restrictions on previous GPGPU approaches, in particular those set by the traditional graphics pipeline and the relative programming interfaces like OpenGL and Direct3D.

We selected NVidia GeForce 8800 and its CUDA platform to design our Smith-Waterman implementation because it is the first available GPU on the market capable of an internal integer representation of data.

**Figure 2:**
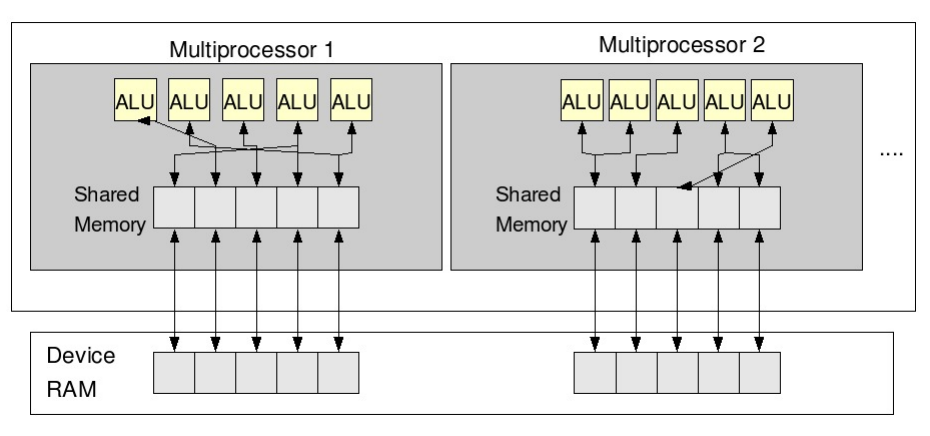
**CUDA architecture.** New CUDA compatible GPUs are implemented as a set of multiprocessors. Each multiprocessor has several ALUs (Arithmetic Logic Unit) that, at any given clock cycle, execute the same instructions but on different data. Each ALU can access (read and write) the multiprocessor *shared memory* and the device RAM.

In CUDA, the GPU is viewed as a compute device suitable for parallel data applications. It has its own device random access memory and may run a very high number of threads in parallel (Figure 2). Threads are grouped in *blocks* and many *blocks* may run in a *grid* of *blocks*. Such structured sets of threads may be launched on a *kernel* of code, processesing the data stored in the device memory. Threads of the same *block* share data through fast shared on-chip memory and can be synchronized through synchronization points. An important aspect of CUDA programming is the management of the memory spaces that have different characteristics and performances:

• Read-write per-thread *registers* (fast, very limited size)

• Read-write per-thread *local memory* (slow, not cached, limited size)

• Read-write per-*block shared memory* (fast, very limited size)

• Read-write per-*grid global memory* (slow, not cached, large size)

• Read-only per-*grid constant memory* (slow but cached, limited size)

• Read-only per-*grid texture memory* (slow but cached, large size)

The proper choice of the memory to be used in each *kernel* depends on many factors such as the speed, the amount needed, and the operations to be performed on the stored data. An important restriction is the limited size of *shared memory*, which is the only available read-write cache. Unlike the CPU programming model, here the programmer needs to explicitly copy data from the *global memory* to the cache (*shared memory*) and backwards. But this new architecture allows the access to memory in a really general way, so both *scatter* and *gather* operations are available. *Gather* is the ability to read any memory cell during the run of the *kernel* code. *Scatter* is the ability to randomly access any memory cell for writing. The unavailability of *scatter* was one of the major limitations of OpenGL when applied to GPGPU applications. The main point in approaching CUDA is that the overall performance of the applications dramatically depends on the management of the memory, which is much more complex than in the CPUs.

## Results and discussion

Exhaustive tests have been performed to compare the implementation of Smith-Waterman in CUDA with:

• the results of W. Liu as reported in his paper [4]. His solution was implemented in OpenGL and was tested on a NVidia GeForce 7900 GPU

• BLAST [1] and SSEARCH [14], running on a 3 GHz Intel Pentium IV processor

• the results of the SIMD implementation by Farrar as reported in his paper [3]. His tests were run on a 2.4 GHz Xeon Core 2 Duo processor.

We have tested our solution on a workstation, having the 2.4 GHz Intel Q6600 processor and two NVidia GeForce 8800 GTX graphic cards. We have measured the performance by running the application both on single and on double GPU configurations. By doubling the computing resources we observed that the overall performance of the application also doubles. This shows that the solution can benefit from a nearly linear speed improvement when adding more graphic boards to the system. It must be mentioned that the Nvidia SLI option, available for multi-GPU systems, is designed for OpenGL. Therefore, SLI must be disabled for CUDA, which requires direct programming of every installed GPU.

### Smith-Waterman in CUDA vs. Liu's implementation

For this test five protein sequences of different length (from 63 to 511 residues) were run against the SwissProt database (Dec. 2006 – 250,296 proteins and 91,694,534 amino acids). The substitution matrix BLOSUM50 with a gap-open penalty of 10 and a gap-extension penalty of 2 were used. The resulting MCUPS for each of the 5 query sequences are shown in Table 1.

**Table 1:** Smith-Waterman in CUDA running on single and double GPU vs. Liu's solution implemented in OpenGL

***Sequence***		SW-Cuda*	SW-Cuda**	***Weiguo Liu***
***Name***	***Length***	***MCUPS***	***MCUPS***	***MCUPS***
*O29181*	63	1849	3561	197
*P03630*	127	1889	3612	317
*P53765*	255	1811	3428	428
*Q8ZGB4*	361	1810	3446	486
*P58229*	511	1795	3353	533

Substitution matrix used: BLOSUM50. Gap-open penalty: 10. Gap-extension penalty: 2.

Database used: SwissProt (Dec. 2006 – 250,296 proteins and 91,694,534 amino acids).

* Smith-Waterman in CUDA running on an NVidia GeForce 8800 GTX

** Smith-Waterman in CUDA running on two NVidia GeForce 8800 GTX

Liu obtained on the same sequences an average of 392.2 MCUPS and a peak of 533 MCUPS. Our solution on a single GPU was completed in a time of 63.5 sec with an average of 1830 MCUPS and a peak of 1889 MCUPS. Our implementation on two GPUs achieved a search time of 33.63 sec with an average of 3480 MCUPS and a peak of 3612 MCUPS. These results indicate that our implementation of Smith-Waterman is up to 18 times faster than that of Liu.

### Smith-Waterman in CUDA vs. BLAST and SSEARCH

For this test we used the same sequences, database and substitution matrix described in the previous paragraph. SSEARCH completed the search in 960 sec with an average of 119.2 MCUPS and a peak of 123 MCUPS. BLAST completed the search in 53.3 sec with an average of 2018 MCUPS and a peak of 2691 MCUPS.

**Figure 3:**
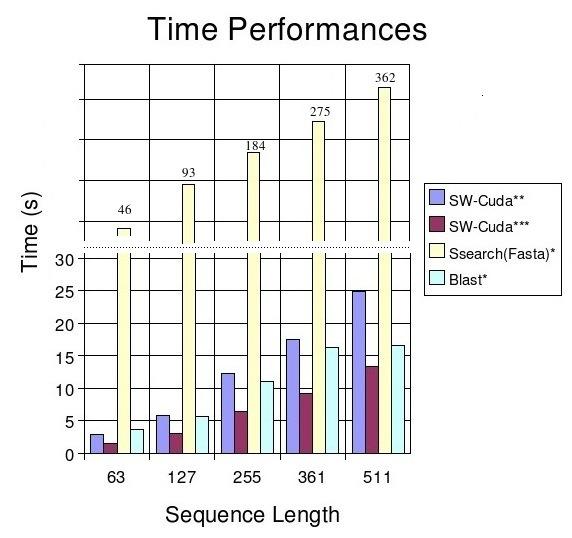
**Smith-Waterman in CUDA running on single and double GPU vs. BLAST and SSEARCH.** Substitution matrix used: BLOSUM50. Gap-open penalty: 10. Gap-extension penalty: 2. Database used: SwissProt (Dec. 2006 – 250,296 proteins and 91,694,534 amino acids). * Smith-Waterman in CUDA running on an NVidia GeForce 8800 GTX ** Smith-Waterman in CUDA running on two NVidia GeForce 8800 GTX

The execution times of our CUDA implementation were up to 30 times faster than SSEARCH and up to 2.4 times faster than BLAST, as shown in Figure 3 and Table 2.

**Table 2:** Smith-Waterman in CUDA running on single and double GPU vs. BLAST and SSEARCH

***Sequence***		SW-Cuda*		SW-Cuda**		***Ssearch(Fasta)***		***Blast***	
***Name***	***Length***	***Time (s)***	***MCUPS***	***Time (s)***	***MCUPS***	***Time (s)***	***MCUPS***	***Time (s)***	***MCUPS***
*O29181*	63	2.98	1849	1.547	3561	46	119	3.7	1488
*P03630*	127	5.88	1889	3.075	3612	93	119	5.7	1948
*P53765*	255	12.31	1811	6.505	3428	184	121	11	2027
*Q8ZGB4*	361	17.44	1810	9.162	3446	275	114	16.3	1936
*P58229*	511	24.89	1795	13.326	3353	362	123	16.6	2691

Substitution matrix used: BLOSUM50. Gap-open penalty: 10. Gap-extension penalty: 2.

Database used: SwissProt (Dec. 2006 – 250,296 proteins and 91,694,534 amino acids).

* Smith-Waterman in CUDA running on an NVidia GeForce 8800 GTX

** Smith-Waterman in CUDA running on two NVidia GeForce 8800 GTX

### Smith-Waterman in CUDA vs. Farrar's implementation

This last test was done running 11 sequences of different length (from 143 to 567 residues) against the SwissProt database (Rel. 49.1 – 208,005 proteins and 75,841,138 amino acids). The substitution matrix is the BLOSUM50 with a gap-open penalty of 10 and a gap-extension penalty of 2.

**Figure 4:**
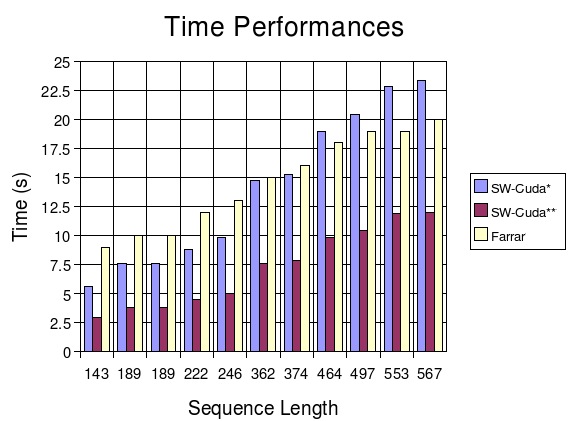
**Smith-Waterman in CUDA running on single and double GPU vs. Farrar's solution**. Substitution matrix used: BLOSUM50. Gap-open penalty: 10. Gap-extension penalty: 2. Database used: SwissProt (Rel. 49.1 – 208,005 proteins and 75,841,138 amino acids). * Smith-Waterman in CUDA running on an NVidia GeForce 8800 GTX ** Smith-Waterman in CUDA running on two NVidia GeForce 8800 GTX

The Farrar's approach is based on the following consideration: for most cells in the alignment matrix, F remains at zero and does not contribute to the value of H. Only when H is greater than *G_init_* + *G_ext_* will F start to influence the value of H. So firstly F is not considered. Then, if required, a second step tries to correct the introduced errors. Farrar's solution completed the search in 161 sec with an average of 1630 MCUPS and a peak of 2045 MCUPS. Our solution running on a single GPU turned in a slightly better time of 154.95 sec with an average of 1783.3 MCUPS and a peak of 1845 MCUPS. On two GPU devices the search was completed in 79.65 sec with an average of 3792.2 MCUPS and a peak of 3575 MCUPS. The search times and resulting MCUPS are shown in Figure 4 and Table 3.

**Table 3:** Smith-Waterman in CUDA running on single and double GPU vs. Farrar's solution

***Sequence***		SW-Cuda*		SW-Cuda**		***Farrar***	
***Name***	***Length***	***Time (s)***	***MCUPS***	***Time (s)***	***MCUPS***	***Time (s)***	***MCUPS***
*P02232*	143	5.59	1845	2.95	3497	9	1149
*P01111*	189	7.59	1796	3.84	3551	10	1367
*P05013*	189	7.59	1796	3.84	3551	10	1367
*P14942*	222	8.84	1812	4.48	3575	12	1338
*P00762*	246	9.85	1802	5.01	3542	13	1369
*P10318*	362	14.71	1775	7.57	3450	15	1746
*P07327*	374	15.28	1766	7.86	3433	16	1691
*P01008*	464	18.96	1765	9.83	3405	18	1864
*P10635*	497	20.39	1758	10.43	3438	19	1892
*P25705*	553	22.83	1747	11.88	3358	19	2105
*P03435*	567	23.32	1754	11.96	3420	20	2045

Substitution matrix used: BLOSUM50. Gap-open penalty: 10. Gap-extension penalty: 2.

Database used: SwissProt (Rel. 49.1 – 208,005 proteins and 75,841,138 amino acids).

* Smith-Waterman in CUDA running on an NVidia GeForce 8800 GTX

** Smith-Waterman in CUDA running on two NVidia GeForce 8800 GTX

Farrar's solution improves its performances on the longer sequences, but on the average, it takes longer than our solution running even on a single GPU. So Smith-Waterman in CUDA is up to 3 times faster than Farrar's implementation.

## Conclusions

Up to now the huge computational power of the GPUs was hampered by the limited programming model of OpenGL which is unsuitable for efficient general-purpose computing.

The results of this work show that the new CUDA compatible graphic cards are now advanced enough to be considered as efficient hardware accelerators for the Smith-Waterman algorithm. High speed can be obtained with the greatest sensitivity. But this work also opens interesting perspectives as similar strategies of acceleration could be applied to a number widely used algorithms in bioinformatics. Thus equal investments in terms of hardware may lead to much better performances. Future work of our team is planned in the direction of accelerating BLAST.

The source files of our Smith-Waterman implementation are available at .

## Methods

### Query-profile

When calculating *H_ij_* the value from the substitution matrix *W(q_i_, d_j_)* is added to *H_i−1_*, *_j−1_*. As suggested by Rognes and Seeberg [9], to avoid the lookup of *W(q_i_, d_j_)* in the internal cycle of the algorithm, we pre-compute a query profile parallel to the query sequence for each possible residue.

**Figure 5:**
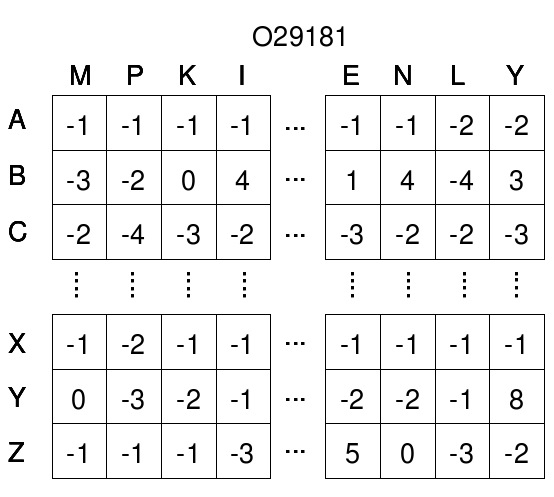
**Query-profile.** Example of query profile for the protein 029181. For each amino acid, a profile row is filled with the scores obtained matching that amino acid with the query residues, based on the given substitution matrix.

The query profile, shown in Figure 5, can be considered as a query-specific substitution matrix computed only once for the entire database. The score for matching symbol A (for alanine) in the database sequence with each of the symbols in the query sequence is stored sequentially in the first matrix row. The scores for matching symbol B are stored in the next row, and so on.

In this way we replace random accesses to the substitution matrix with sequential ones to the query profile. This solution exploits the cache of the GPU *texture memory* space where the query profile is stored.

### Smith-Waterman in CUDA

A great number of parallel threads have to be launched simultaneously to fully exploit the huge computational power of the GPU. The strategy adopted in our implementation in CUDA was to make each GPU thread compute the whole alignment of the query sequence with one database sequence. As explained in the section about the CUDA programming model, the threads are grouped in a *grid* of *blocks* when running on the graphics card. In order to make the most efficient use of the GPU resources the computing time of all the threads in the same *grid* must be as near as possible. For this reason we found it was important to pre-order the sequences of the database in function of their length. So when running, the adjacent threads will need to align the query sequence with two database queries having the nearest possible sizes.

Following is the optimal configuration of threads allowing for the best performance:

• number of threads per *block*: 64

• number of *blocks*: 450

• total number of threads per *grid*: 28800

The ordered database is stored in the *global* GPU memory, while the query-profile is saved into a *texture*. For each alignment the matrix is computed column by column in order parallel to the query sequence. To compute a column we need all the H and E values from the previous one. We store them in the *local memory* of the thread. More precisely, we use two buffers: one for the previous values and one for the newly computed ones. At the end of each column we swap them and so on. *Local memory* is not cached, so it is very important to choose the right access pattern to this space. The GPU is able to read and write up to 128 bits of the *local memory* with a single instruction. So each thread reads at once four H and four E values (16 bits long) from the loading buffer plus the respective four values from the profile. It computes the four results for the new column, then it stores them in the storing buffer. To fully take advantage of the memory bandwidth of the graphics card we package the profile in the *texture*, saving four successive values (always minor than 255) into the four bytes of a single unsigned integer. Thus, each thread can gather all the data needed to compute four cells of the alignment matrix with only two read instructions (one from the local buffer and one from the *texture*).

**Figure 6:**
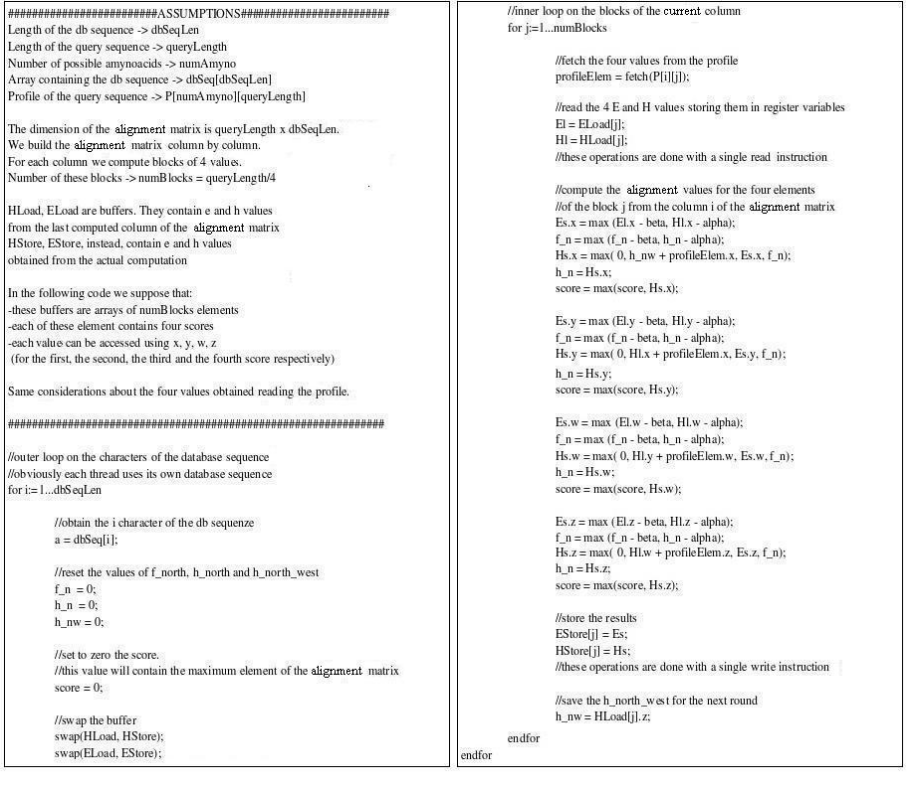
***Kernel* pseudo code**. Each thread executes this code on a different database sequence. The pseudo-code for the Smith-Waterman implementation is made up of the outer loop, which cycles on the database sequence characters, followed by the inner loop, which does the basic dynamic programming calculations.

**Figure 7:**
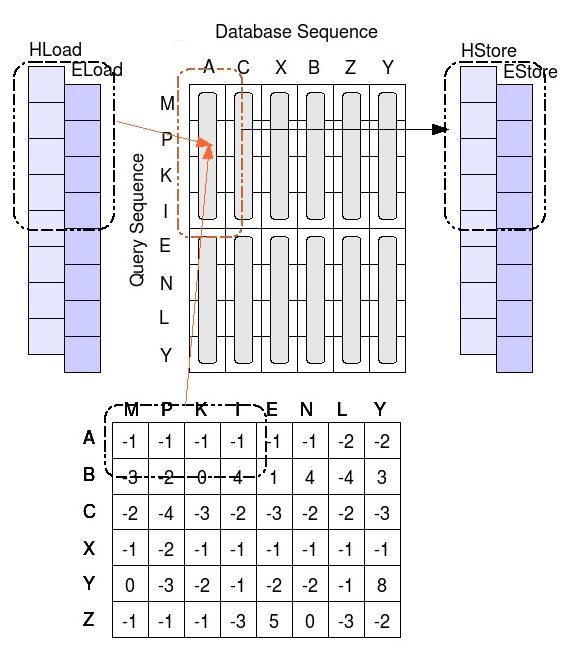
**Smith-Waterman in CUDA functioning.** Each thread gathers four E and H values from the load buffer (first read operation) and four values from the profile (second read operation: the four values are packaged in a single unsigned integer of the query-profile). The Smith-Waterman algorithm is then applied to these data and the results are saved in the storing buffer (a single write operation). The alignment also requires two supplementary values: an f_north and an h_north. In this case, there is no need to save an entire column, but only two temporary numbers updated at each cell computation.

Figure 6 has the pseudo code of the *kernel* executed by each thread, while Figure 7 shows the interactions between the *local memory* buffers and the query-profile to compute the alignment matrix.

Before running Smith-Waterman, the implementation automatically detects the number of computational resources available. A dynamic load balancing is achieved according to the number of devices and their computational power. The database is split in the same number of segments as the number of GPUs. Each GPU then computes the alignment of the query with one database segment. The size of the segment depends upon the power of that GPU. The speed of each device is computed after every alignment. A new partitioning of the database is done for the successive query on the base of a weighted average of the performances detected during previous runs. Pre-fixed weights are used for the first run.

## List of abbreviations used

CTM – Close To Metal

CUDA – Compute Unified Device Architecture

SIMD – Single Instruction, Multiple Data

GPU – Graphics Processing Unit

GPGPU – General Purpose computing on Graphics Processing Unit

CPU – Central Processing Unit

CUPS – Cells Updates Per Second

SSE – Streaming SIMD Extensions

## Competing interests

The authors declare that they have no competing interests.

## Authors' contributions

SAM coordinated the study, designed the strategies of parallelization and the architecture of the solution and contributed to writing the manuscript. GV provided the idea for the study, contributed to discussions, analysed the results and contributed to revising of the manuscript. Both authors read and approved the final manuscript.
